# Perioperative dexmedetomidine effects on delirium in elderly patients after noncardiac surgery: A retrospective propensity score analysis

**DOI:** 10.3389/fphar.2025.1578233

**Published:** 2025-05-23

**Authors:** Hong-Wei Wang, Qin-Jun Chu, Ze-Fei Zhu, Ming Cheng, Ze-Ping Li, Liang Zang, Long He, Lin-Na Chen, Qian He, Jian-Jun Yang, Han-Wen Gu

**Affiliations:** ^1^ Department of Anesthesiology, Pain and Perioperative Medicine, The First Affiliated Hospital of Zhengzhou University, Zhengzhou, China; ^2^ Department of Anesthesiology and Perioperative Medicine, Zhengzhou Central Hospital Affiliated to Zhengzhou University, Zhengzhou, China; ^3^ Department of Interventional Radiology, The First Affiliated Hospital of Zhengzhou University, Zhengzhou, China; ^4^ Department of Medical Information, The first Affiliated Hospital of Zhengzhou University, Zhengzhou, China; ^5^ Department of Anesthesiology, The First Hospital of Jilin University, Changchun, China; ^6^ Department of Anesthesiology and Perioperative Medicine, The First Affiliated Hospital of Xinxiang Medical University, Weihui, China

**Keywords:** dexmedetomidine, elderly patients, intensive care unit, delirium, propensity score analysis postoperative delirium, postoperative nausea and vomiting, propensity score matching

## Abstract

**Background:**

Delirium is a complex syndrome with limited pharmacological treatment options, whereas non-pharmacological prevention strategies warrant further investigation. Dexmedetomidine, an α2-adrenergic receptor agonist commonly used for sedation and analgesia, has shown potential anti-inflammatory effects that may contribute to delirium prevention. We conducted a retrospective PSM analysis to evaluate the effectiveness of dexmedetomidine in preventing postoperative delirium in elderly ICU patients undergoing noncardiac surgery.

**Methods:**

A retrospective analysis was conducted, including patients undergoing noncardiac surgeries after surgery. The main outcome was the 7-day incidence of delirium. Secondary outcomes included the length of hospital stay, postoperative nausea and vomiting, and postoperative complications. Propensity score matching and regression models were utilized to adjust for confounders and to investigate associations between the use of dexmedetomidine and outcomes.

**Results:**

A total of 19,899 patients were included, and 3,169 pairs were matched after propensity score matching. After matching, the incidence of postoperative delirium was 8.68% in the cohort with perioperative dexmedetomidine (test group) and 17.80% in the cohort without dexmedetomidine (control group), *p* < 0.001. The numerical rating scale in the test group was significantly decreased (mean ± SD, 2.4 ± 0.9 vs. 2.6 ± 0.8, *p* < 0.001). Hypotension (14.86% vs. 14.04%, *p* < 0.001) was increased, whereas hypertension (10.67% vs. 13.13%, *p* < 0.001) and tachycardia (16.81% vs. 10.71%, *p* < 0.001) were decreased in the test group.

**Conclusion:**

Perioperative infusion of dexmedetomidine may reduce the incidence of delirium in elderly patients after noncardiac surgery.

## 1 Introduction

Delirium is a syndrome characterized by acute disturbances in attention, awareness, and cognition, and it can manifest in various psychomotor subtypes (hyperactive, hypoactive, and mixed) and often lacks effective pharmacological treatments (Wilson et al., 2020). A recent literature work emphasizes that perioperative delirium is underdiagnosed and undertreated, partly due to knowledge and practice gaps among clinicians (Ragheb et al., 2023). However, there is a lack of evidence supporting pharmacological prophylaxis for the prevention of delirium (Swarbrick and Partridge, 2022). Preventive strategies such as cognitive pre-habilitation, perioperative geriatric assessment, multidisciplinary care, dexmedetomidine, and multimodal analgesia have been suggested, but further research is needed to determine their efficacy in reducing delirium incidence (Wilson et al., 2020; Ron and Deiner, 2024).

Dexmedetomidine, an α2-adrenergic receptor agonist, is widely used in clinical and research settings due to its sedative, analgesic, and anxiolytic properties (Chen et al., 2024). Evidence suggests that dexmedetomidine may also possess anti-inflammatory effects, which may contribute to the prevention of delirium through the suppression of inflammatory signaling and cytokine production (Yamazaki et al., 2022). Recent systematic reviews and network meta-analyses indicate that dexmedetomidine significantly reduces the incidence of postoperative delirium compared to other sedatives (Huang et al., 2024; Li et al., 2021).

Given the potential of dexmedetomidine in delirium prevention and the strength of propensity score matching (PSM) in controlling for bias in observational studies (Chen et al., 2022), we conducted a retrospective PSM analysis to assess the effectiveness of dexmedetomidine in preventing postoperative delirium (POD) in elderly ICU patients after noncardiac surgery. Our hypothesis posits that dexmedetomidine administration can decrease the incidence of POD in this population.

## 2 Methods

### 2.1 Study design and patient selection

In this single-center retrospective observational study, patient data were obtained from the First Affiliated Hospital of Zhengzhou University, ethical approval number (2024-KY-1062-001). This study was conducted and reported in accordance with strengthening the reporting of observational studies in epidemiology (STROBE) guidelines (von Elm et al., 2007).

Elderly patients who underwent noncardiac surgery were included in this study. Patients who were admitted to an ICU after surgery were included. Patients aged below 65 years, who underwent neurosurgeries, who were applied non-general anesthesia, or who were unconscious before surgeries were excluded. The included patients were divided into two groups (control and test groups) based on the usage of perioperative dexmedetomidine.

### 2.2 Outcome measurements

The primary outcome was the 7-day incidence of POD. Secondary outcomes included 3-day postoperative nausea and vomiting (PONV) and in-hospital mortality. Postoperative complications included 7-day incidences of bradycardia, tachycardia, hypotension, hypertension, and hypoxemia. POD was assessed twice daily at 8 a.m. and 8 p.m. using the confusion assessment method (CAM) or the confusion assessment method for the intensive care unit (CAM-ICU) as appropriate. Patients in the ICU were assessed using CAM-ICU, and those who had moved out of the ICU were assessed using CAM. The presence of PONV was determined by physicians during visits. Bradycardia and tachycardia were defined as heart rates lower than 60 times per minute or greater than 100 times per minute, which persisted for at least 1 min. Patient pain intensities were evaluated using the numerical rating scale (NRS). Hypertension and hypotension were defined as systolic blood pressure greater than 140 mmHg or lower than 90 mmHg. Hypoxemia was defined as a blood oxygen level lower than 90%.

Patient characteristics included age, sexuality, ASA scores, preoperative comorbidities, type of surgeries, perioperative infusion volume, blood loss, and usage of perioperative and postoperative analgesics, perioperative vasopressors, and antihypertensive drugs. Perioperative sedatives included dexmedetomidine, propofol, remimazolam, etomidate, desflurane, esketamine, and ciprofol. Postoperative analgesics included dexmedetomidine, hydromorphone, esketamine, palonosetron, butorphanol, tropisetron, dexamethasone, dezocine, dolasetron, flurbiprofen, ketorolac tromethamine, nalbuphine, oxycodone, sufentanil, azasetron hydrochloride, betamethasone, metoclopramide, prednisone, pentazocine, and eptazocine.

### 2.3 Statistical analysis

All statistical analyses were performed using RStudio (RStudio 2023.06.0 Build 421, R version 4.4.1).

Descriptive data are presented as the medians (interquartile ranges) for continuous variables and frequencies (%) for categorical variables. Categorical variables were compared between groups using the chi-square tests. For continuous variables, Shapiro–Wilk’s test was used to test their normality, and the unpaired t-test and Mann–Whitney U test were used for normally and non-normally distributed data, respectively.

Propensity score analysis was performed using R package “MatchIt” (version 4.5.5) (Ho et al., 2011). Propensity scores were calculated through a generalized linear model. Greedy nearest neighbor matching was performed at 1:1 ratio with replacement, no action was taken to optimize an overall criterion, and each match was selected without considering the other matches that may occur subsequently. The caliper was set to 0.2 of the standard deviation of the logit of the propensity score, as recommended by a previous work (Austin, 2011). All patient characteristics were used for calculation of the propensity scores. Additional regression models were fitted to evaluate the effect of perioperative dexmedetomidine. A subgroup analysis by surgery type was conducted to assess differences in dexmedetomidine’s effect on postoperative delirium.

## 3 Results

**FIGURE 1:**
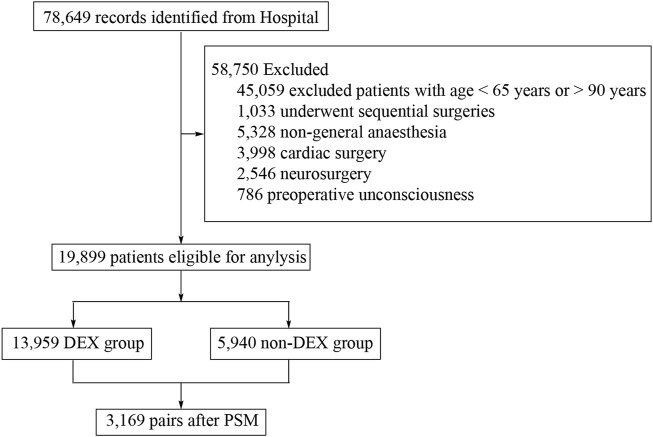
Study flowchart.

Records of 78,649 patients who underwent surgeries between Jan. 01, 2021, and Oct. 31, 2024, were screened for eligibility, from which 45,059 were excluded because patients were aged below 65 years or beyond 90 years; 1,033 patients underwent sequential surgeries; 5,328 were excluded for using non-general anesthesia during surgery; 3,998 and 2,546 patients who underwent cardiac surgeries and neurosurgeries were excluded. Another 786 patients who were unconscious before surgery were excluded. A total of 19,899 patients were included in the analysis. A total of 3,169 pairs of patients were matched after PSM. The flowchart of patient selection is presented in Figure 1.

Patient characteristics are summarized in Table 1. A total of 13,959 (70.15%) patients used dexmedetomidine during surgery. Before PSM, most confounders were imbalanced between the groups. After matching, patients in the test group had more male patients (57.05% vs. 54.37%, *p* = 0.032). For preoperative comorbidities, patients in the test group had lower prevalence of hypertension (30.86% vs. 33.23%, *p* = 0.034) and kidney diseases (10.44% vs. 13.32%, *p* < 0.001) and had more patients with chronic liver diseases (10.76% vs. 8.84%, *p* = 0.01). The two groups had different infusion volumes (2,000, 750–3,100 with dexmedetomidine vs. 1,600, 750–2,850 without dexmedetomidine, *p* < 0.001). For perioperative medication, patients in the test group used more esketamine (17.99% vs. 15.30%, *p* = 0.004), less desflurane (28.43% vs. 31.59%, *p* = 0.006), more ciprofol (38.56% vs. 33.20%, *p* < 0.001), more remimazolam (39.44% vs. 34.05%, *p* < 0.001), more vasopressors (67.78% vs. 64.78%, *p* = 0.012), and more antihypertensive drugs (47.02% vs. 35.22%, *p* < 0.001). Postoperative analgesics also differed in dezocine (16.28% vs. 14.26%, *p* = 0.025). The types of surgeries were not balanced as the categorical chi-squared test indicated a *p-*value lower than 0.001.

**TABLE 1:** Patient characteristics.

Variables		Before PSM			After PSM		
		Control (N = 5,940)	Test (N = 13,959)	*P*	Control (N = 3,169)	Test (N = 3,169)	*P*
Age (years)		71 [68–75]	70 [67–74]	<0.001	71 [67–75]	71 [68–75]	0.11
Male, n (%)		3,906 (65.76)	7,159 (51.29)	<0.001	1,723 (54.37)	1,808 (57.05)	0.032
Weight		65 [58–72.5]	65 [56.6–70]	<0.001	64 [56–71]	64 [55–70]	0.211
Preoperative comorbidities, n (%)							
Hypertension		2,668 (44.92)	4,838 (34.66)	<0.001	1,057 (33.35)	978 (30.86)	0.034
Cancer		2,172 (36.57)	5,052 (36.19)	0.616	1,232 (38.88)	1,187 (37.46)	0.245
Chronic heart disease		513 (8.64)	1,019 (7.30)	0.001	421 (13.28)	388 (12.24)	0.214
Chronic liver disease		605 (10.19)	1,519 (10.88)	0.145	280 (8.84)	341 (10.76)	0.01
Type II diabetes		1,689 (28.43)	685 (4.91)	<0.001	391 (12.34)	381 (12.02)	0.701
Pneumonia		529 (8.91)	1,816 (13.01)	<0.001	339 (10.70)	303 (9.56)	0.134
Kidney disease		501 (8.43)	1,462 (10.47)	<0.001	422 (13.32)	331 (10.44)	<0.001
ASA, n (%)							
1		277 (4.66)	820 (5.87)	<0.001	165 (5.21)	168 (5.30)	0.391
2		3,811 (64.16)	9,773 (70.01)		2,094 (66.08)	2,080 (65.64)	
3		1,784 (30.03)	3,239 (23.20)		879 (27.74)	875 (27.61)	
4		68 (1.14)	127 (0.91)		31 (0.98)	46 (1.45)	
Perioperative variables, n (%), median [IQR]							
Esketamine		1,575 (26.52)	3,100 (22.21)	<0.001	485 (15.30)	570 (17.99)	0.004
Propofol		599 (10.08)	22 (0.16)	<0.001	19 (0.60)	15 (0.47)	0.492
Desflurane		2,578 (43.40)	2,111 (15.12)	<0.001	1,001 (31.59)	901 (28.43)	0.006
Ciprofol		2,550 (42.93)	4,802 (34.40)	<0.001	1,052 (33.20)	1,222 (38.56)	<0.001
Remimazolam		1,122 (18.89)	5,989 (42.90)	<0.001	1,079 (34.05)	1,250 (39.44)	<0.001
Etomidate		5,232 (88.08)	10,756 (77.05)	<0.001	2,493 (78.67)	2,544 (80.28)	0.113
Vasopressors		3,706 (62.39)	10,681 (76.52)	<0.001	2,053 (64.78)	2,148 (67.78)	0.012
Antihypertensive		2,236 (37.64)	7,467 (53.49)	<0.001	1,116 (35.22)	1,490 (47.02)	<0.001
Infusion volume (mL)		1,350 [500–1825]	2,450 [1,350–3,250]	<0.001	1,600 [750–2,850]	2,000 [750–3,100]	<0.001
Blood loss (mL)		50 [20–100]	50 [20–100]	0.387	50 [20–100]	50 [20–100]	0.344
Postoperative analgesics, n (%)							
Dexmedetomidine		29 (0.49)	119 (0.85)	0.006	17 (0.54)	29 (0.92)	0.076
Hydromorphone		1598 (26.90)	5,428 (38.89)	<0.001	959 (30.26)	1,011 (31.90)	0.158
Esketamine		237 (3.99)	796 (5.70)	<0.001	140 (4.42)	118 (3.72)	0.162
Palonosetron		1,465 (24.66)	4,707 (33.72)	<0.001	856 (27.01)	859 (27.11)	0.932
Butorphanol		80 (1.35)	206 (1.48)	0.484	57 (1.80)	52 (1.64)	0.629
Tropisetron		35 (0.59)	139 (1.00)	0.005	31 (0.98)	42 (1.33)	0.195
Dexamethasone		41 (0.69)	120 (0.86)	0.222	29 (0.92)	24 (0.76)	0.49
Dezocine		713 (12.00)	2,807 (20.11)	<0.001	452 (14.26)	516 (16.28)	0.025
Dolasetron		35 (0.59)	239 (1.71)	<0.001	35 (1.10)	52 (1.64)	0.066
Flurbiprofen		619 (10.42)	1710 (12.25)	<0.001	363 (11.45)	367 (11.58)	0.875
Ketotromethamine^a^		96 (1.62)	233 (1.67)	0.788	51 (1.61)	64 (2.02)	0.221
Nalbuphine		37 (0.62)	121 (0.87)	0.076	22 (0.69)	15 (0.47)	0.248
Other^b^		36 (0.61)	120 (0.86)	0.063	27 (0.85)	26 (0.82)	0.89
Type of surgeries, n (%)							
Facial		408 (6.87)	2,523 (18.07)	<0.001	390 (12.31)	585 (18.46)	<0.001
General		2,468 (41.55)	6,169 (44.19)		1,516 (47.84)	1,544 (48.72)	
Gynecologic		106 (1.78)	598 (4.28)		79 (2.49)	85 (2.68)	
Orthopedic		745 (12.54)	3,232 (23.15)		638 (20.13)	406 (12.81)	
Urinary		2,213 (37.26)	1,437 (10.29)		546 (17.23)	549 (17.32)	

^a^ Ketorolac tromethamine.

^b^ Metoclopramide, oxycodone, sufentanil, azasetron hydrochloride, betamethasone, prednisone, pentazocine, and eptazocine were grouped as “other” due to low frequency (<0.02%).

**FIGURE 2:**
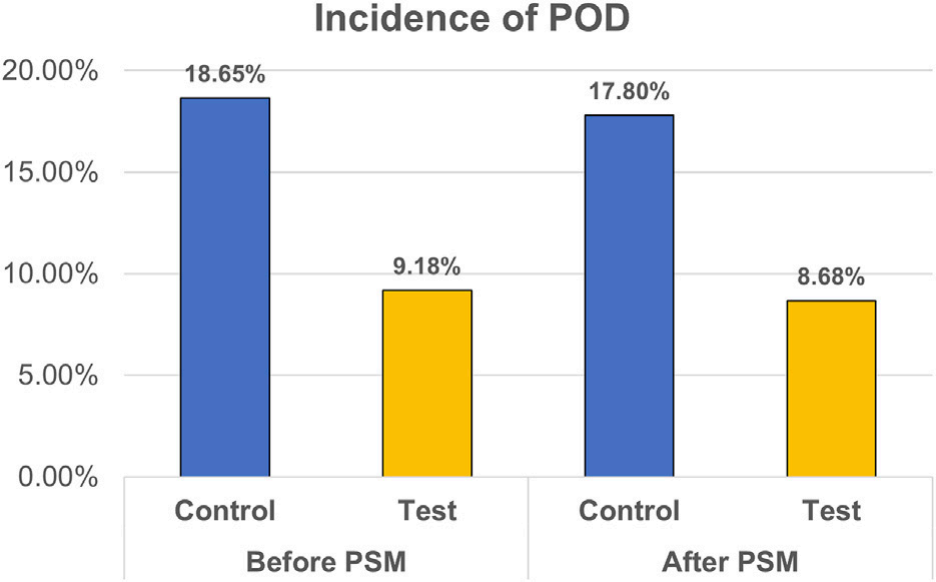
Incidence of POD before and after PSM.

The analysis of postoperative outcomes is shown in Table 2, and the main result is presented in Figure 2. For patients in the control group, 1,108 out of 5,940 (18.65%) patients experienced POD with average duration of 4.5 days. The incidence of POD was significantly lower in the test group before PSM (*p* < 0.001). Incidences of tachycardia (10.85% in test group vs. 15.78% in control group, *p* < 0.001), hypertension (9.99% vs. 12.90%, *p* < 0.001), and PONV (9.9% vs. 12.17%, *p* < 0.001) were decreased in the test group, whereas incidences of hypotension (14.97% vs. 13.67%, *p* < 0.001) and bradycardia (21.12% vs. 16.36%, *p* = 0.021) were increased. Patient pain intensities were reduced (mean ± SD, 2.4 ± 0.9 vs., 2.6 ± 0.8, *p* < 0.001).

**TABLE 2:** Outcome measurements.

Outcomes	Before PSM				After PSM			
	Control (N = 5,940)	Test (N = 13,959)	OR (95%CI)	*P*	Control (N = 3,169)	Test (N = 3,169)	OR (95%CI)	*P*
POD, n (%)	1,108 (18.65)	1,282 (9.18)	0.44 (0.40–0.49)	<0.001	564 (17.80)	275 (8.68)	0.43 (0.37–0.50)	<0.001
POD durations*	4.5 ± 1.0	4.5 ± 0.9	1.01 (0.77–1.34)	0.935	4.5 ± 1.1	4.5 ± 1.0	1.04 (0.90–1.21)	0.168
PONV, n (%)	723 (12.17)	1,382 (9.90)	0.80 (0.71–0.90)	<0.001	386 (12.18)	307 (9.69)	0.75 (0.64–0.89)	<0.001
NRS	2.6 ± 0.8	2.4 ± 0.9	0.85 (0.83–0.88)	<0.001	2.6 ± 0.8	2.4 ± 0.9	0.87 (0.84–0.91)	<0.001
Hypotension, n (%)	812 (13.67)	2090 (14.97)	1.34 (1.22–1.48)	<0.001	445 (14.04)	471 (14.86)	1.39 (1.22–1.59)	<0.001
Hypertension, n (%)	766 (12.90)	1,395 (9.99)	0.66 (0.59–0.73)	<0.001	416 (13.13)	338 (10.67)	0.73 (0.63–0.84)	<0.001
Bradycardia, n (%)	972 (16.36)	2,948 (21.12)	1.13 (1.02–1.26)	0.021	506 (15.97)	659 (20.80)	1.07 (0.93–1.23)	0.37
Tachycardia, n (%)	938 (15.79)	1,515 (10.85)	0.74 (0.66–0.83)	<0.001	501 (15.81)	372 (11.74)	0.79 (0.68–0.92)	0.003
Hypoxemia, n (%)	486 (8.18)	1,137 (8.15)	1.00 (0.87–1.14)	0.957	262 (8.27)	249 (7.86)	0.98 (0.81–1.17)	0.795
Mortality, n (%)	56 (0.94)	155 (1.11)	1.43 (0.97–2.13)	0.072	27 (0.85)	40 (1.26)	1.51 (0.89–2.54)	0.124

* POD durations were analyzed through the subset of patients with POD.

Effects of perioperative dexmedetomidine were calculated through linear regression and logistic regression for continuous variables and categorical variables, respectively.

**FIGURE 3:**
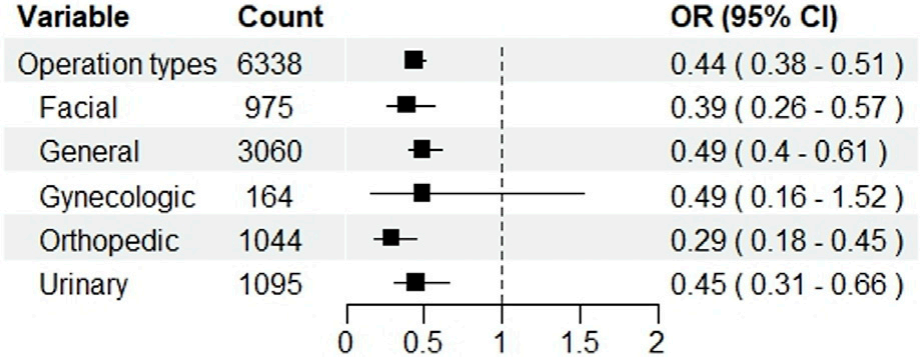
Subgroup analysis by surgery type.

After PSM, 275 out of 3,169 (8.68%) patients in the test group and 564 (17.80%) in the control group experienced POD (*p* < 0.001). In the test group, the incidence of hypotension (14.86% vs. 14.04%, *p* < 0.001) was increased, whereas the incidences of PONV (9.69% vs. 12.18%, *p* < 0.001), tachycardia (11.74% vs. 15.81%, *p* = 0.003), and hypertension (10.67% vs. 13.13%, *p* < 0.001) were decreased. NRS was lowered in the test group (2.4 ± 0.9 vs. 2.6 ± 0.8, *p* < 0.001). Subgroup analysis showed that dexmedetomidine significantly reduced the risk of postoperative delirium across most surgical types, with pooled ORs ranging from 0.29 to 0.49. Significant reductions were observed in facial, general, orthopedic, and urinary surgeries, whereas no clear benefit was seen in gynecologic surgeries. The result is presented in Figure 3.

## 4 Discussion

In this retrospective propensity score analysis, perioperative dexmedetomidine was found to be preventive for POD in elderly patients after noncardiac surgery. Moreover, dexmedetomidine decreases postoperative pain intensity and hypertension, whereas it increases the risk of hypotension.

The incidence of POD in patients without dexmedetomidine was 18.65% in this study, which was in line with previous studies (Su et al., 2016; Zhu et al., 2023). Dexmedetomidine, as a selective alpha-2 adrenoreceptor agonist, has emerged as a promising pharmacological intervention for the prevention and treatment of delirium due to its ability to decrease sympathetic nervous system activity and the release of excitatory neurotransmitters such as noradrenaline in the brain (Veronese et al., 2024). Many studies have reported supporting evidence. Liu et al. (2024) found that dexmedetomidine applied as local anesthesia adjuvant reduced POD in elderly patients after hip surgeries. A RCT reported by Hu et al. (2021) also reported decreased POD in elderly patients undergoing open transthoracic esophagectomy. Li et al. also conducted a RCT concluding that dexmedetomidine halved the risk of POD for elderly patients after major noncardiac surgeries (Li et al., 2020).

Recent research expanded the understanding of the mechanisms of dexmedetomidine, showing that it mitigated postoperative delirium through complementary neuroprotective mechanisms. First, it attenuates neuroinflammation by suppressing microglial NF-κB signaling and reducing pro‐inflammatory mediators such as TNF-α and IL-6 in the brain (Cai et al., 2025; Fondeur et al., 2022). Concurrently, dexmedetomidine may act on the cholinergic anti-inflammatory pathway to inhibit systemic TNF release (Huang et al., 2020; Jacob et al., 2023). Finally, dexmedetomidine may preserve blood–brain barrier (BBB) integrity by upregulating tight-junction proteins and limiting BBB permeability​, thereby preventing peripheral cytokines from invading the central nervous system (Hu et al., 2022).

We also found that perioperative infusion of dexmedetomidine also decreased patient pain intensity by a mean value of 0.2 of NRS. This was consistent with several previous reports (Su et al., 2016; Lee et al., 2018). The overall pain intensity in the first postoperative day was relatively lower in this study, which may be caused by applied postoperative patient-controlled intravenous anesthesia. Other secondary outcomes, including patient hospital stays, and postoperative nausea and vomiting, were familiar for both groups.

For common postoperative complications, perioperative dexmedetomidine increased hypotension while decreasing hypertension in this study. Deng et al. (2022) recently reported an increased risk of hypotension with dexmedetomidine compared with other sedatives. The decrease of hypertension was also reported by an earlier RCT (Su et al., 2016). Bradycardia and hypoxemia were not affected in this study, which was supported by another PSM study (Zhao et al., 2024). Furthermore, in-hospital mortality was not affected by perioperative dexmedetomidine.

This study has several advantages. First, the sample size of 19,899 patients was relatively larger than that in other studies, and the PSM balanced patient baseline characteristics and minimized the effect of confounders, which provided evidence as strong as randomized-controlled trials, as patient baseline characteristics were balanced after propensity scoring, and the effect of observed confounders were minimized. Second, multiple perioperative and postoperative analgesics were included as confounders and were balanced between the groups of our study, and this provided a more general insight into the effect of dexmedetomidine.

Our study has limitations as well. First, in PSM, it is assumed that all confounders have been included in the analysis; however, in a retrospective study, it is likely that important parameters were missing. The dose, duration, time, and routes of infusion of dexmedetomidine have been reported to be influential on postoperative delirium (Niu et al., 2023; Qu et al., 2023). Second, the data were collected from a single center over a relatively long period of time. The change of anesthesia protocol may be influential to the result. The results may not be representative of other cohorts, as research regarding patients who underwent cardiac surgeries often report contradictory results.

## 5 Conclusion

Perioperative dexmedetomidine can reduce the risk of postoperative delirium in elderly patients after noncardiac surgery.

## Data Availability

The datasets presented in this article are not readily available due to ethical and privacy concerns of publishing patient data. Requests to access the datasets should be directed to Hong-Wei Wang, whw0526@163.com.

## Abbreviations

ASA, the American Society of Anesthesiologists; BBB, blood–brain barrier; CAM, confusion assessment method; CAM-ICU, confusion assessment method for the intensive care unit; ICU, intensive care unit; IL-6, interleukin-6; NF-κB, nuclear factor kappa B; NRS, numerical rating scale; POD, postoperative delirium; PONV, postoperative nausea and vomiting; PSM, propensity score matching; RCT, randomized controlled trial; STROBE, Strengthening the Reporting of Observational Studies in Epidemiology; TNF-α, tumor necrosis factor-alpha.
